# Supplementary material for: Improving the Recyclability of an Epoxy Resin through the Addition of New Biobased Vitrimer
Source: Polymers (Basel). 2023 Sep 12;15(18):3737. doi: 10.3390/polym15183737 (PMC10537514; doi:10.3390/polym15183737)
Supplement: Supplementary file 1 [file polymers-15-03737-s001.zip › polymers-2551871-SM.pdf]

Supplementary Materials

Table S1. TGA results obtained for samples: $T_{30\%}$ and final residual mass %.

Sample	$T_{30\%}$ (°C)	Final Residue wt%
**VESOV**	377.7	11.7
**VESOV+ER (30 wt%)**	360.5	14.2
**VESOV+ER (50 wt%)**	360.6	17.4
**VESOV+ER (70 wt%)**	355.3	16.8
**VSB+ER**	354.1	15.8

The most significant signals of the FTIR spectra of the starting reagents used, as well as the VSB are assigned.

Vanillin (cm^{-1}): 3650–3200 (ν, OH), 2850 (ν, O=C-H), 1670 (ν, C=O), 1590–1450 (ν, C-C arom), 1250 (ν_{sim}, C-O-C), 1150–1000 (ν, C-O(H)), 850–600 (δ_{oop}, 3 bands H-Carom).

1,4-butandiamine (cm^{-1}): 3400–3250 (ν, N-H), 2950 (ν_{as}, H-Csp^3), 2850 (ν_{sim}, H-Csp^3), 1625–1500 (δ, N-H), 1200–1025 (ν_{sim}, C-N), 800 (δ, N-H), 730 (ϒ, CH_2).

VSB (cm^{-1}): 3600–3250 (ν, OH), 3080–3030 (ν, H-Carom), 2975 (ν_{as}, H-Csp^3), 2825 (ν_{sim}, H-Csp^3), 1655 (ν, C=N), 1590–1450 (ν, C-C arom), 1250 (ν_{sim}, C-O-C), 1150–1000 (ν, C-O(H)), 875–750 (δ_{oop}, 3 bands H-Carom).

The following figures **S1 and S2** show the assignments to the displacement peaks obtained by ^1H-NMR of BDA and VAN, as well as the different signals obtained: vanillin, 1,4-butandiamine and VSB.

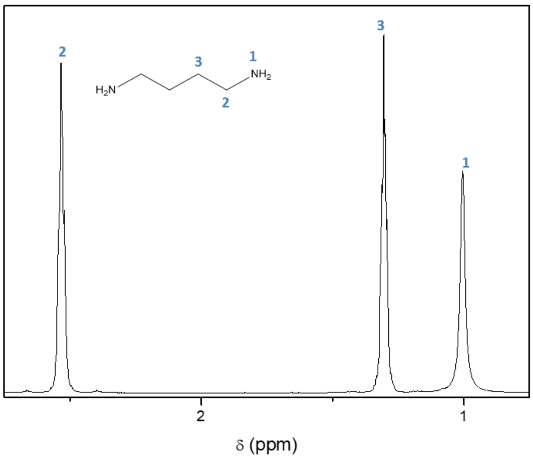

Figure S1. ^1H-NMR spectrum of 1,4-butandiamine.

^1H-NMR 1,4-butandiamine ($CDCl_3$, 600 MHz) δ (ppm): 2.53 (t, J = 7.4 Hz, 4H, CH_2, H-2), 1.30 (t, J = 7.4 Hz, 4H, CH_2, H-3), 1.00 (s, 4H, NH_2).

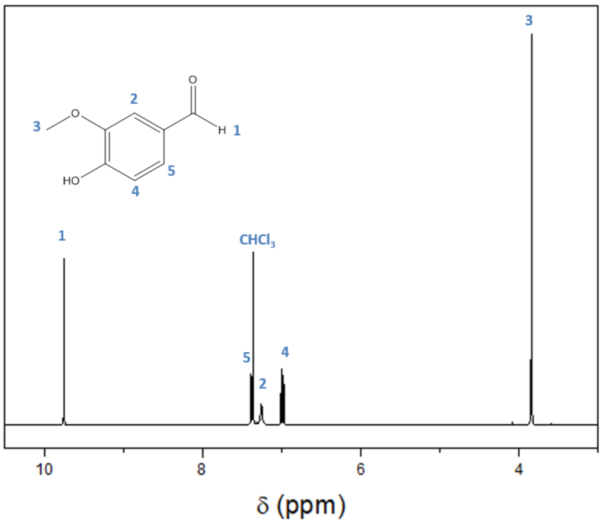

Figure S2. ^{1}H-NMR spectrum of vanillin.

^{1}H-NMR Vanillin ($CDCl_3$, 600 MHz) δ (ppm): 9.74 (s, 1H, $H_{Aldehyde}$), 7.36 (dd, J = 7.7, 1.3 Hz, 1H, H-5_{arom}), 7.19 (dd, J = 1.3 Hz, 1H, H-2_{arom}), 6.98 (dd, J = 7.7 Hz, 1H, H-4_{arom}), 3.85 (s, 3H, OMe).

^{1}H-NMR VSB ($CDCl_3$, 600 MHz) δ (ppm): 9.83 (s, 1H, OH), 8.17 (s, 2H, H-2), 7.41 (dd, J = 8.4, 1.7 Hz, 2H, H-8_{arom}), 7.08 (dd, J = 1.7 Hz, 2H, H-4_{arom}), 6.91 (dd, J = 8.4 Hz, 2H, H-7_{arom}), 3.93 (s, 6H, OMe), 3.69 (t, J = 7.4 Hz, 4H, CH_2, H-2), 1.77 (t, J = 7.4 Hz, 4H, CH_2, H-1).

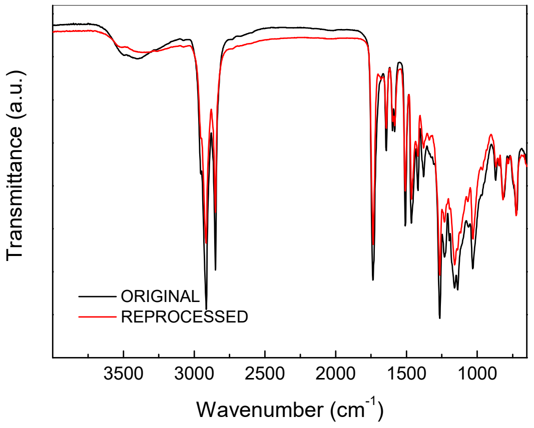

VSB-ESO

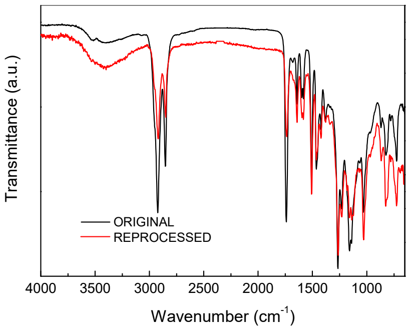

VSB-ESO+30% ER

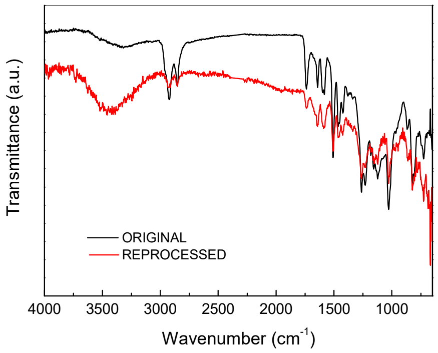

VSB-ESO+50% ER

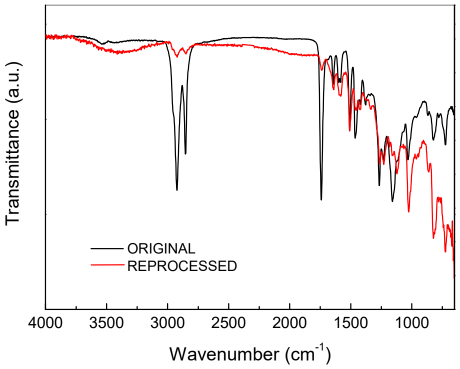

VSB-ESO+70% ER

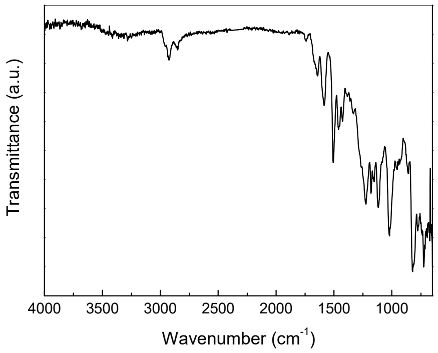

VSB-ER

Figure S3. FTIR spectra of pre- (black) and post-reprocessed (red) sample.
